# Enzyme replacement therapy attenuates disease progression in two Japanese siblings with mucopolysaccharidosis type VI: 10-Year follow up

**DOI:** 10.1016/j.ymgmr.2017.08.007

**Published:** 2017-09-14

**Authors:** Mahoko Furujo, Motomichi Kosuga, Torayuki Okuyama

**Affiliations:** a Department of Pediatrics, National Okayama Medical Center, , 1711-1, Tamasu, Kita-ku, Okayama 701-1192, Japan; b Center for Lysosomal Storage Diseases, National Center for Child Health and Development, 2-10-1 Okura, Setagaya-ku, Tokyo 157-8535, Japan

**Keywords:** Case report, Deficient *N*-acetylgalactosamine 4-sulfatase, Enzyme replacement therapy, Galsulfase, Glycosaminoglycan, Mucopolysaccharidosis type VI, ASB, *N*-acetylgalactosamine 4-sulfatase, ECHO, echocardiography, ERT, enzyme replacement therapy, fT4, free thyroid hormone, GAG, glycosaminoglycan, GH, growth hormone, IGF-1, insulin-like growth factor 1, MPS, mucopolysaccharidosis, NR, normal range, rh, recombinant human, ROM, range of motion, TSH, thyroid stimulating hormone, 6MWT, 6-minute walk test

## Abstract

Early initiation of enzyme replacement therapy (ERT) has demonstrated clinical benefit in patients with mucopolysaccharidosis type VI (MPS VI), a progressive, multisystem autosomal recessive lysosomal disorder caused by *N*-acetylgalactosamine-4-sulphatase (ASB) deficiency and the consequent accumulation of glycosaminoglycan. A previous case report highlighted that 3 years of ERT with recombinant human ASB (galsulfase) was well tolerated and effective in two Japanese siblings with MPS VI who initiated ERT at 5.6 years and 6 weeks of age, respectively. This report describes 10-year follow-up data from these two siblings who continued ERT with weekly infusions of galsulfase 1 mg/kg. Ten years of ERT was well tolerated, and the older sibling reached puberty. He had typical MPS VI phenotypic features, but exhibited significant improvement in shoulder range of motion and had largely unchanged hearing and cardiac function. His skeletal deformity remained unchanged. In contrast, in the younger sibling, typical symptoms of MPS VI, including progressive dysmorphic facial features, hepatosplenomegaly, and hearing impairment were largely absent. Her joint mobility was preserved, although skeletal deformity, including claw-hand deformity, was observed. Both siblings had progressive corneal clouding. The observations in these two patients suggest that early ERT initiated in newborns can be well tolerated and effective in preventing or slowing MPS VI disease progression, but is limited in terms of its effects on bone symptoms. For this, new approaches or bone-targeting treatments would be necessary.

## 1 Introduction

Mucopolysaccharidosis type VI (MPS VI; or Maroteaux–Lamy syndrome) is a very rare autosomal recessive lysosomal disorder. Estimates of incidence range from 0.36 to 1.30 per 100,000 live births and varies between countries and ethnic populations [1], [2]. The true incidence of MPS VI is likely higher because of the lack of newborn screening programs and the difficulty in diagnosing the disease [2].

The pathology underlying MPS VI is a mutation in the *N*-acetylgalactosamine 4-sulfatase (arylsulfatase B, or ASB; EC 3.1.6.12) gene. This mutation reduces or eliminates ASB function which, in turn, impairs the stepwise degradation of dermatan sulfate [3]. The consequent accumulation of glycosaminoglycan (GAG) in lysosomes in a wide range of tissues causes short stature, dysostosis multiplex, typical coarse facial features (ie, macroglossia, a broad nose, a broad nasal bridge, large rounded cheeks, and thick lips), corneal clouding, and cardiac and pulmonary manifestations, which often require clinical interventions, and reduce patients' functioning and lifespan [3], [4]. Pharmacological treatment of MPS VI is limited to enzyme replacement therapy (ERT) with galsulfase, which is recommended as first-line therapy by international management guidelines for MPS VI [4]. Galsulfase (recombinant human ASB; rhASB; Naglazyme^®^) was approved for treating MPS VI in Japan in 2008.

Starting ERT in patients with MPS VI at a young age has generated much recent interest [5], [6], [7], [8], given that patients present with MPS VI signs and symptoms from the first year of age and usually by 2 years of age [2], [4]. Findings from studies of MPS VI using animal models have indicated that starting ERT with galsulfase at birth, before the development of MPS VI signs and symptoms, leads to better long-term outcomes [9], [10]. Further, findings from recent case reports suggest that starting ERT at a young age has a clear benefit in preventing or slowing disease progression of MPS VI [5], [6], [7], [8]. Starting ERT at a young age may also have a positive effect on cardiac abnormalities [11], which are common in patients with MPS VI and an important cause of morbidity and mortality [4]. Although these findings are encouraging, there is a lack of long-term efficacy and safety data regarding the use of galsulfase as ERT for MPS VI in previous clinical studies [12], [13], especially in pediatric patients who start ERT at a very young age (< 1 year) and continue to take ERT indefinitely [6], [8]. In addition, few endocrine assessment data have been published for pediatric patients with MPS VI treated with ERT [14].

In a previous case report, we detailed the safety and efficacy findings associated with 3 years of ERT with galsulfase in a Japanese brother and sister with MPS VI who initiated ERT at 5.6 years and 6 weeks of age, respectively [5]. Here, we present 10-year follow-up data for these siblings, both of whom continued ERT with galsulfase.

## 2 Materials and methods

### 2.1 Patient information and treatment

This is a case report of two Japanese siblings with MPS VI who received 10 years of ERT between November 2006 and May 2017 at National Okayama Medical Center in Japan.

The siblings were born to consanguineous Japanese parents, both of whom had a missense mutation p.Y85H (c.252T > C). Likewise, both siblings had the homozygous missense mutation Y85H in the ASB gene [5]. The demographic and clinical characteristics of the siblings have been described previously [5]. In brief, Sibling 1 was diagnosed with MPS VI when he was 3.3 years of age and started ERT when he was 5.6 years of age in November 2006. Sibling 2, the younger sister of Sibling 1, was diagnosed with MPS VI after her delivery and started ERT at 6 weeks of age in May 2007. The parents received genetic counselling while the mother was pregnant with Sibling 2.

ERT comprised recombinant human ASB galsulfase (BioMarin Pharmaceuticals Inc., San Rafael, USA) diluted with physiological saline solution (Japanese Pharmacopoeia) infused at 1 mg/kg/week over a 4-hour period.

Parental consent for inclusion of patient data and photographs for this report was obtained.

### 2.2 Outcome measures

Endurance was evaluated using the 6-minute walk test (6MWT) every 12 weeks after starting ERT for Sibling 1 and every 12 weeks from 30 months after birth for Sibling 2. Neither sibling was evaluated for endurance at the start of ERT.

Urinary GAG concentration was determined as the concentration of uronic acid normalized for creatinine (mg/g creatinine) and was measured using the carbazole reaction [15], [16] at a central laboratory (SRL Medisearch Inc., Tokyo, Japan) at the start of ERT, every 12 weeks in the first 24 months, every 24 weeks from 25 to 84 months, and every 6 months thereafter.

Height and weight were measured before ERT and every 12 weeks thereafter. Tanner stage was assessed. Joint range of motion (ROM) tests, skeletal X-rays, and audiology examinations were performed at the start of ERT and every 24 weeks thereafter. Active joint ROM was measured by goniometry, and included shoulder (flexion and abduction), elbow (flexion), hip (flexion and abduction), and knee (flexion) assessments.

Endocrine assessments were performed at 3, 9, and 10 years after starting ERT, including insulin-like growth factor 1 (IGF-1) and estradiol (for Sibling 2), assessed at a central laboratory (SRL Medisearch Inc., Tokyo, Japan); testosterone (for Sibling 1), free thyroid hormone (fT4), and thyroid stimulating hormone (TSH) were assessed locally.

Tanner development stage was confirmed 10 years after starting ERT.

Standard 12-lead electrocardiography was performed at the start of ERT and every 12 weeks during ERT. Cardiac structure and function were evaluated by echocardiography (ECHO, two-dimensional and M-mode).

Safety evaluations included continuous monitoring of adverse events and periodic assessment of clinical laboratory/vital signs (every 12 weeks) and physical examinations. Serum was collected every 6 weeks and sent to a central laboratory (BioMarin Pharmaceutical Inc.) for evaluation of immunoglobulin G anti-rhASB antibody levels as determined by antibody assays.

## 3 Results

### 3.1 Exposure and safety

By May 2017, Sibling 1 and Sibling 2 had been on ERT for 10.5 and 10.0 years, respectively. Since the previous report in 2011 [5], neither sibling has experienced any infusion-associated reactions or drug-related adverse events.

Both siblings developed antibodies to rhASB; Sibling 1 after 6 weeks of treatment, and Sibling 2 after 80 weeks of treatment. As of August 2016, both siblings had a positive response (each at a dilution factor of 65,610).

### 3.2 Outcome measures

#### 3.2.1 Endurance

There were insufficient 6MWT data because the children were not always able to follow the instructions correctly. The 6MWT in Sibling 1 was 305 m and 420 m at 3 and 10 years after ERT, respectively, which was approximately half the normal reference distance (adjusted for height and age) at both time points [17]. The 6MWT in Sibling 2 was 655 m at 10 years after ERT and was not different from the normal reference distance [17].

#### 3.2.2 Uronic acid

**Fig. 1:**
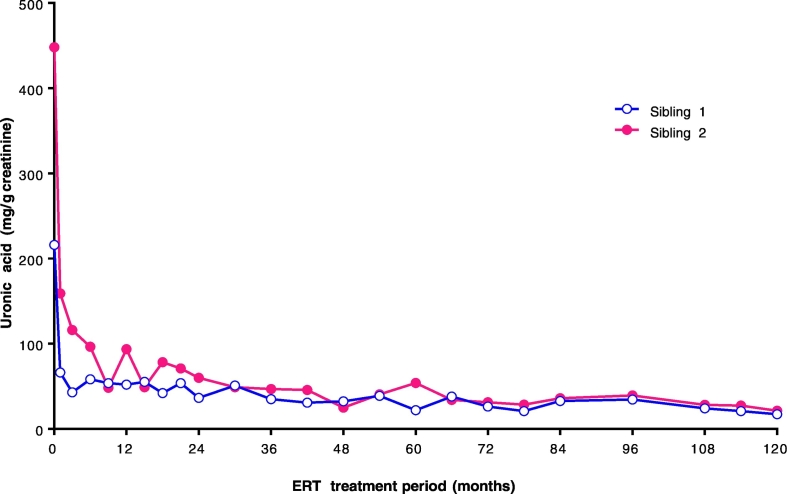
Uronic acid levels for each sibling.

Urinary uronic acid at 120 months after starting ERT was 17.1 mg/g creatinine (normal range [NR], 10.1 ± 3.1 mg/g at 16 years of age) in Sibling 1 and 21.3 mg/g creatinine (NR, 19.4 ± 6.8 mg/g at 10 years of age) in Sibling 2. Urinary uronic acid concentrations did not reach the relevant age-dependent NR in either sibling, but did decline rapidly after starting ERT and remained below 40 mg/g creatinine for the last 3 years (Fig. 1).

#### 3.2.3 Clinical signs and symptoms

**Fig. 2:**
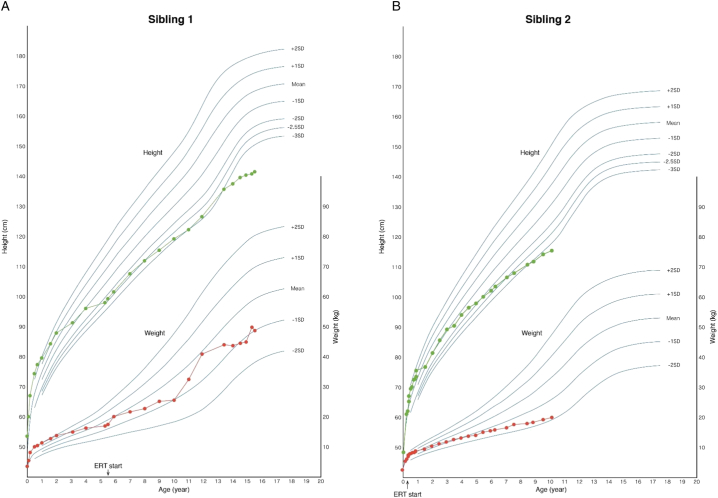
Growth charts of height and weight of Sibling 1 (A) and Sibling 2 (B) compared with aged-matched data from a national survey of Japanese children in 2000 [28].

Height and weight of both siblings increased over time (Fig. 2); however, both measurements were lower than the corresponding age-related Japanese norms. For Sibling 1 at 10 years after starting ERT, the height was 141.4 cm (more than three standard deviations below the age-appropriate mean) and the weight was 48.7 kg (one standard deviation below the mean). For Sibling 2 at 10 years after starting ERT, the height was 115.5 cm (more than three standard deviations below the mean) and the weight was 20.0 kg (more than one standard deviation below the mean).

Sibling 1 was at Tanner Stage 2 at 13.3 years of age (his voice was starting to change) and at Tanner Stage 3 at 15.5 years of age. Sibling 2 was at Tanner Stage 1 at 10 years of age.

**Fig. 3:**
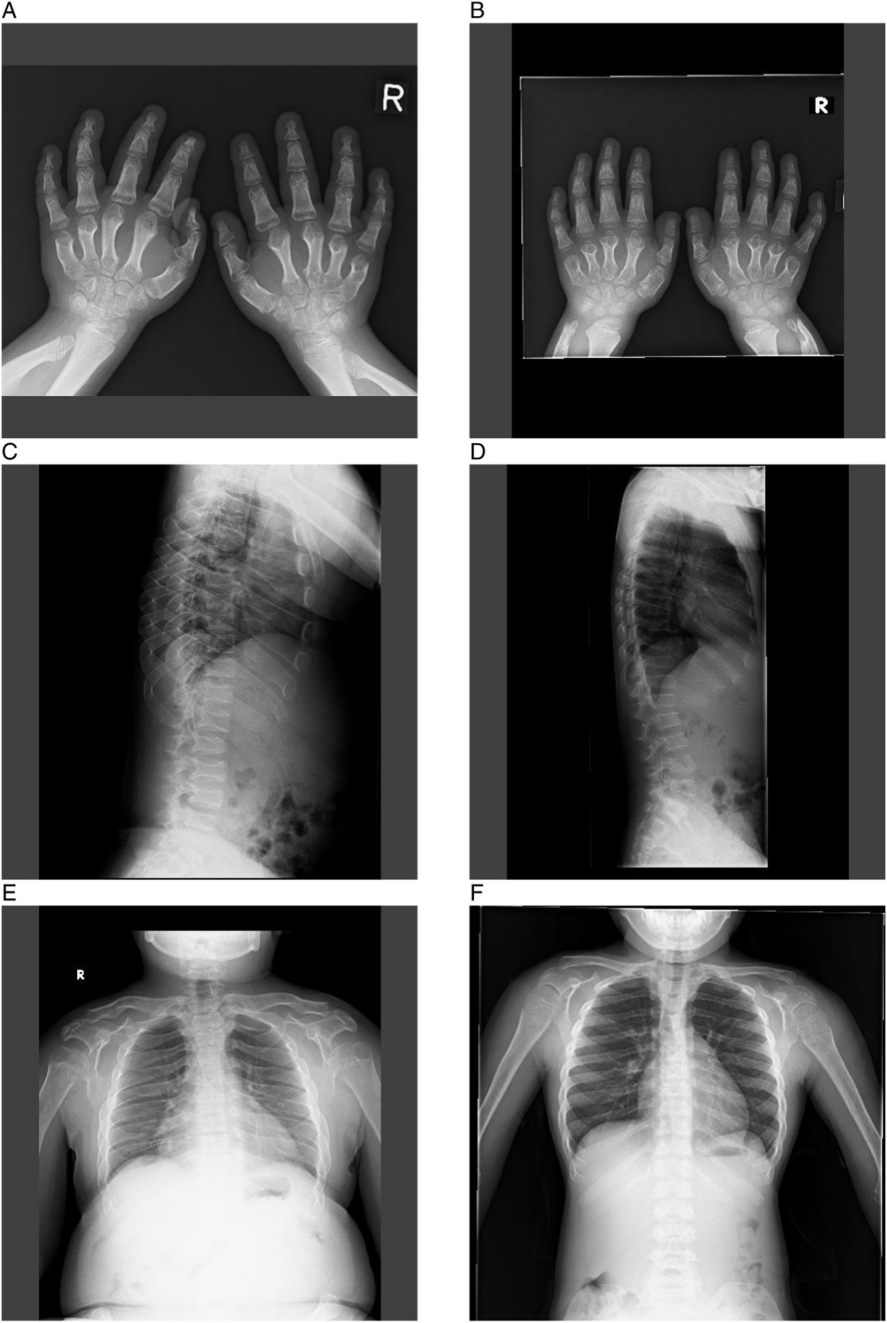
Radiographs of hands and spinal column of Sibling 1 (A, C, E) and Sibling 2 (B, D, F) at 10.2 and 9.7 years, respectively, after starting enzyme replacement therapy.

Radiography as at February 2017 for Sibling 1 (15 years of age) and January 2017 for Sibling 2 (9 years of age) was characteristic of MPS VI (Fig. 3). The hand radiographs showed point-shaped metacarpal bones in both siblings. Regarding spines, Sibling 1 presented defective development of the vertebral bodies with abnormal egg-shape and anterior beaking. Sibling 2 also showed egg-shaped vertebral bodies with anterior beaking, before starting and during ERT. Oar-shaped ribs were observed in both siblings.

**Fig. 4:**
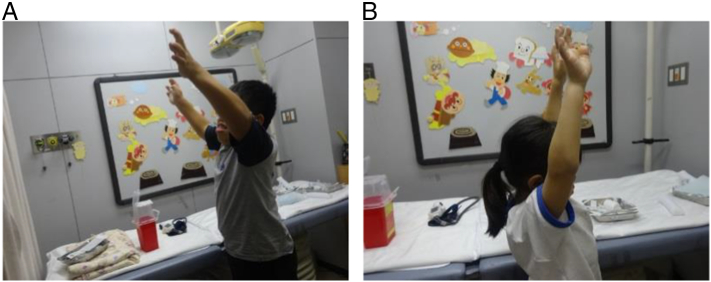
Photograph showing shoulder joint range of motion of Sibling 1 (A) and Sibling 2 (B) at 10.0 and 9.5 years, respectively, after starting enzyme replacement therapy.

For joint ROM, both siblings experienced improvement in shoulder ROM for 10 years after starting ERT (Fig. 4; Table 1). In Sibling 1, right shoulder flexion increased from 105 degrees before ERT to 130 degrees after 10 years of ERT, and left shoulder flexion increased from 115 degrees to 140 degrees. In Sibling 2, right shoulder flexion increased from 100 degrees before ERT to 160 degrees after 10 years of ERT, and left shoulder flexion increased from 90 degrees to 160 degrees. As the joint ROM at baseline of Sibling 2 was measured at 6 weeks of age, the measured ROM may underestimate the true ROM, because of muscular tension during crying. The limited points of available joint ROM data did not allow us to identify when the peak occurred.

**Table 1:** Comparison of clinical signs and symptoms between Sibling 1 and Sibling 2 at baseline, 3 years, and 10 years after starting of ERT.

Clinical signs and symptoms	Sibling 1			Sibling 2		
	Baselinea,^b^	3 yr after ERT^b^	10 yr after ERT	Baselinea,^b^	3 yr after ERT^b^	10 yr after ERT
Facial appearance	Coarse face	Coarse face	Coarse face	Non-MPS VI characteristic	Non-MPS VI characteristic	Non-MPS VI characteristic
Hand	Claw-hand deformity	Claw-hand deformity	Claw-hand deformity	Normal	Mild claw-hand deformity	Claw-hand deformity
Joint ROM (degrees)						
Shoulder flexion	R: 105 L: 115	R: 125 L: 150	R: 130 L: 140	R: 100 L: 90	R: 180 L: 180	R: 160 L: 160
Shoulder abduction	R: 85 L: 85	R: 100 L: 110	R: 90 L: 125	R: 100 L: 90	R: 180 L: 180	R: 160 L: 160
Elbow flexion	R: 125 L: 130	R: 120 L: 130	R: 155 L: 155	R: 120 L: 120	R: 145 L: 140	R: 160 L: 165
Hip flexion	R: 135 L: 135	R: 95 L: 90	R: 110 L: 120	R: 120 L: 120	R: 110 L: 110	R: 110 L: 110
Hip abduction	R: 60 L: 60	R: 40 L: 35	R: 25 L: 25	R: 60 L: 60	R: 50 L: 50	R: 50 L: 50
Knee flexion	R: 140 L: 140	R: 140 L: 135	R: 130 L: 150	R: 120 L: 120	R: 150 L: 155	R: 145 L: 150
Gait	Toe-walking	Toe-walking	Toe-walking	Normal	Normal	Normal
Aortic valve	Normal	Thickening appeared at 12 months of ERT and remained unchanged	Thickening	Normal	Thickening appeared at 6 months of ERT and then became normal	Mild thickening
Mitral valve	Mild insufficiency	Thickening; Level 1 regurgitation	Thickening; Level 1 regurgitation	Slightly abnormal regurgitation	Normal	Normal
Tricuspid valve	Mild insufficiency	Thickening; slight regurgitation	Thickening; Level 1 regurgitation	Slightly abnormal regurgitation	Normal	Slightly abnormal regurgitation
Audiology	Moderate hearing loss (around 45 dB)	Normal ABR with hearing improvements after ERT (hearing declined at 30 months of ERT, but was improved with ear tube insertion; hearing improved further at 36 months of ERT)	Normal (20 dB for both ears)	Normal	Hearing tests remained normal at most assessments (except at 24 months of ERT when ABR test was abnormal and left ear 35 dB V-wave slightly extended)	Normal (20 dB for both ears)
Ophthalmology	Moderate corneal clouding, glaucoma	Moderate corneal clouding	Moderate corneal clouding	Normal	Mild corneal clouding	Moderate corneal clouding
Size of liver and spleen	Liver: 517 mL Spleen: 139 mL	Liver: 780 mL^c^ Spleen: 120 mL^c^	Liver: 1017 mL Spleen: 180 mL	Liver: 97 mL Spleen: No data	Liver: 380 mL Spleen: 65 mL	Liver: 475 mL Spleen: 86 mL
Antibody to rhASB	Negative	590,490 DF	65,610 DF	Negative	7290 DF	65,610 DF

Abbreviations: ABR = auditory brainstem response; dB = decibel; DF = dilution factor; ERT = enzyme replacement therapy; L = right, R = right, rhASB = recombinant human arylsulfatase B; ROM = range of motion; yr = years.

a Baseline: before or at start of ERT.

b Data published previously in Furujo et al. 2011 [5].

c Data at 4 years after start of ERT.

**Fig. 5:**
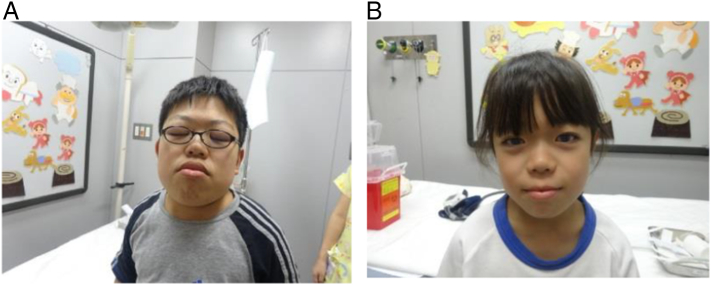
Photograph showing facial appearance of Sibling 1 (A) and Sibling 2 (B) at 10.1 and 9.6 years, respectively, after starting enzyme replacement therapy.

Sibling 1 had a characteristic MPS VI facial appearance at 10.1 years of age, whereas Sibling 2 did not present typical MPS VI facial appearance at 9.6 years of age (Fig. 5).

Sibling 1 had an umbilical hernia until 13 years of age. Neither sibling has experienced significant progression in cardiac symptoms (Table 1, ECHO not shown). Progressive corneal clouding was observed in both siblings (Table 1). Other assessments, including hand deformity, gait, audiology, and size of liver/spleen are summarized in Table 1.

#### 3.2.4 Endocrine hormones

In Sibling 1, the IGF-1 concentration increased from 188 ng/mL at 2.8 years after starting ERT (at 8.4 years of age) to 312 ng/mL at 9 years after starting ERT (at 14.5 years of age), and was 301 ng/mL at 9.6 years after starting ERT (at 15.2 years of age). The NR of IGF-1 for adolescent boys is 141 to 552 ng/mL [18]. Sibling 1's testosterone concentration was 3.88 ng/mL after 9 years of ERT and 1.77 ng/mL after 9.6 years. The NR of testosterone is 2.2 to 6.2 ng/mL for middle adolescence (equivalent to Tanner stage 3) [18]. Sibling 1's fT4 and TSH concentrations were 1.09 ng/mL (NR, 0.70 to 1.70 ng/mL [18]) and 1.90 μIU/mL (NR, 0.50 to 4.30 μIU/mL [18]), respectively, after 9.6 years of ERT.

In Sibling 2, the IGF-1 concentration increased from 63 ng/mL at 2.3 years after starting ERT (at 2.4 years of age) to 80 ng/mL at 8.5 years after starting ERT (at 8.5 years of age), and 107 ng/mL at 9.1 years after starting ERT (at 9.2 years of age). The NR of IGF-1 for girls before puberty is 89 to 357 ng/mL [18]. Sibling 2's estradiol concentration was < 10 pg/mL (NR, 0 to 31 ng/mL [19]) after 8.5 and 9.1 years of ERT, and the fT4 and TSH concentrations were 1.15 ng/mL (NR, 0.70 to 1.70 ng/mL [18]) and 3.84 μIU/mL (NR, 0.50 to 4.30 μIU/mL [18]), respectively, after 9.1 years of ERT.

## 4 Discussion

This case report provides unique clinical evidence regarding the 10-year efficacy and safety of ERT with galsulfase in two siblings with MPS VI, a very rare autosomal recessive lysosomal disorder. We observed that infusion of ERT for up to 10 years was well tolerated and prevented further disease progression.

Our findings of growth are consistent with the recently published MPS VI growth data that suggest starting ERT early is important to increase growth potential [20]. Sibling 1 showed progression in Tanner staging while receiving ERT and reached puberty. Although he experienced a slight height growth spurt at puberty, his height was more than three standard deviations below normal. This finding is consistent with those from another report of patients with MPS VI who started ERT at a young age [14]. Sibling 2 has not yet reached puberty; her height was more than three standard deviations below normal. Patients with MPS VI are more likely to undergo precocious puberty, caused by hydrocephalus, spinal cord disorders, and compressive neuropathies. However, due to dysostosis multiplex, it is difficult to measure bone age and to detect precocious puberty early, making it necessary to carefully monitor further growth.

Our 10-year follow-up report suggests that starting ERT at a young age is associated with an acceptable safety profile. In agreement with our observations, data from three clinical studies have shown that ERT for up to 5 years resulted in sustained improvements in endurance and an acceptable safety profile; however, the number of patients (*n* = 4) who had 5 full years of ERT was small [12]. In another report, longer-term ERT (6.2 to 11.2 years) was found to be well tolerated in 9 Taiwanese patients with MPS VI who were aged 1.4 to 21.1 years at the start of treatment [13]. Three patients in this cohort experienced mild hypersensitivity reactions that were readily managed using routine premedication therapy [13].

As MPS VI is a progressive disease, delaying its progression, especially that of joint movement, and slowing the rate of deterioration can significantly benefit patients. Notably, Sibling 2 (who started ERT at 6 weeks of age) has not presented with the characteristic MPS VI facial appearance, and has developed fewer clinical manifestations than Sibling 1. This finding is in agreement with those from other reports of patients with MPS VI who started ERT at a young age [5], [6], [7], [8].

However, despite the early start of ERT, radiography in Sibling 2 who started ERT at 6 weeks of age revealed bone deformity characteristic of MPS VI. Even though it is difficult to conclude based on this case observation without comparison with the natural history of the disease in untreated patients, the growth and radiological findings suggest that the ERT's effect on height and bone would have limitations. Recent reports suggest that TNF-α and other inflammatory cytokines may be involved in the pathophysiology of bone and cartilage disease in MPS, and that treatments targeting these mechanisms may be effective for alleviating skeletal symptoms [21], [22]. In a randomized, placebo-controlled pilot study, the TNF-α inhibitor adalimumab reduced bodily pain compared with placebo in a small number of subjects [21]. In an animal study, pentosan polysulfate, an oral medication with anti-inflammatory and pro-chondrogenic properties, reduced inflammatory markers in serum and tissue, and led to marked improvements in motility and other variables [22]. However, further research would be necessary to develop such new approaches aimed at improving bone symptoms via interruption of inflammatory pathways. In addition, the development of bone-targeted ERT may be expected, similar to the manner in which asfotase alfa has been associated with improved skeletal findings in infants and young children with life-threatening hypophosphatasia [23].

This 10-year follow-up shows that the siblings have experienced no significant progression in cardiac symptoms, suggesting that starting ERT at a young age may have a positive effect on cardiac function. In contrast, the results from a previous long-term observational study (53 patients with pre- and post-ERT ECHO data) suggest that ERT neither resolves nor prevents cardiac valve problems [24]. Similarly, a survey study found that there was neither significant improvement nor worsening of cardiac valve outcomes based on the number of patients with or without valve abnormalities; however, the aortic regurgitation rate was somewhat increased (25/31 at follow-up vs 16/31 at baseline) [25]. In another study, ERT in children younger than 12 years was found to reduce intraventricular septal hypertrophy and prevent progression of cardiac valve abnormalities [11]. Clearly, further evidence is needed to determine whether early initiation of ERT improves cardiac function.

We found that the concentration of endocrine hormones (IGF-1, fT4, and TSH) were maintained at near-normal levels in both siblings after approximately 10 years of ERT. There is relatively little information in the literature about endocrine hormone levels in patients with MPS VI, although growth hormone (GH)/IGF-1 deficiency or resistance has been described [14], [26]. In addition, Gardner reported growth and endocrine assessment data on final adult height and endocrine complications in children with Hurler Syndrome, following hematopoietic stem cell transplantation [27]. In this cohort, 8 of 13 patients tested had evidence of high GH concentrations and 1 patient had GH deficiency. Other notable findings included: adrenal and thyroid function were normal; 11 patients were pubertal or postpubertal; 2 female patients had pubertal failure requiring intervention; and all male patients had spontaneous and complete puberty (3 had reduced testicular volumes) [27]. The lack of information regarding endocrine outcomes in patients with MPS VI may be because endocrine assessment is not required as part of postmarketing surveillance for galsulfase. We suggest that such surveillance would be worthwhile to help identify potentially important findings and encourage the collection of endocrine data for patients with MPS VI.

In conclusion, 10 years of ERT with galsulfase in two young Japanese siblings with MPS VI was well tolerated and effective in preventing disease progression. Continued monitoring of these patients will provide important information regarding the effects of long-term ERT on subsequent growth and fertility. Our findings highlight that starting galsulfase in the neonatal period, during early manifestation of the disease, is crucial for effective management of MPS VI, although ERT is limited in terms of its effects on bone symptoms. For these symptoms, new approaches or bone-targeting treatments may be necessary.

## Funding support

This research did not receive any specific grant from funding agencies in the public, commercial, or not-for-profit sectors.

Medical writing assistance was provided by Hiroko Ebina, BPharm, Ph, MBA and Mark Snape, MB BS, CMPP of ProScribe – Envision Pharma Group, and was funded by BioMarin Pharmaceutical Inc. ProScribe's services complied with international guidelines for Good Publication Practice (GPP3).

## Role of the sponsor

BioMarin Pharmaceutical Inc., the manufacturer/licensee of galsulfase, supplied galsulfase used in this research until launch, and contributed towards the medical writing costs for this report. BioMarin Pharmaceutical Inc. was involved in the decision to submit this case report for publication, but did not have a role in the data collection or interpretation.

## Role of contributors

All authors participated in the interpretation of the patients' course, and in the drafting, critical revision, and approval of the final version of the manuscript. MF, the clinical investigator, was involved in the research design and collected patient data.

## Conflicts of interest

The authors received the writing assistance from BioMarin Pharmaceutical Inc. Otherwise, MF has no conflicts of interest to declare. MK reported grants and personal fees from Anges Inc.; personal fees and non-financial support from BioMarin; grants and personal fees from Sanofi K.K. and Shire Japan K.K. TO reported support from Sanofi K.K. and Sumitomo Dainippon Pharma Co., Ltd. outside the submitted work.
